# Utilizing Lean Software Methods To Improve Acceptance of Global eHealth Initiatives: Results From the Implementation of the Basic Emergency Care App

**DOI:** 10.2196/14851

**Published:** 2021-05-26

**Authors:** Christian Rose, Taylor Nichols, Daniel Hackner, Julia Chang, Steven Straube, Willem Jooste, Hendry Sawe, Andrea Tenner

**Affiliations:** 1 Department of Emergency Medicine Stanford University School of Medicine Palo Alto, CA United States; 2 Department of Emergency Medicine University of California, San Francisco San Francisco, CA United States; 3 Department of Emergency Medicine University of Cape Town/Stellenbosch University Cape Town South Africa; 4 Department of Emergency Medicine Muhimbili University of Health and Allied Sciences Dar es Salaam United Republic of Tanzania

**Keywords:** lean, eHealth, emergency, global health, app development, decision support, primary survey, mHealth, Africa, Tanzania, low- and middle income countries, LMIC

## Abstract

**Background:**

Health systems in low- and middle-income countries face considerable challenges in providing high-quality accessible care. eHealth has had mounting interest as a possible solution given the unprecedented growth in mobile phone and internet technologies in these locations; however, few apps or software programs have, as of yet, gone beyond the testing phase, most downloads are never opened, and consistent use is extremely rare. This is believed to be due to a failure to engage and meet local stakeholder needs and the high costs of software development.

**Objective:**

World Health Organization Basic Emergency Care course participants requested a mobile point-of-care adjunct to the primary course material. Our team undertook the task of developing this solution through a community-based participatory model in an effort to meet trainees’ reported needs and avoid some of the abovementioned failings. We aimed to use the well-described Lean software development strategy—given our familiarity with its elements and its ubiquitous use in medicine, global health, and software development—to complete this task efficiently and with maximal stakeholder involvement.

**Methods:**

From September 2016 through January 2017, the Basic Emergency Care app was designed and developed at the University of California San Francisco. When a prototype was complete, it was piloted in Cape Town, South Africa and Dar es Salaam, Tanzania—World Health Organization Basic Emergency Care partner sites. Feedback from this pilot shaped continuous amendments to the app before subsequent user testing and study of the effect of use of the app on trainee retention of Basic Emergency Care course material.

**Results:**

Our user-centered mobile app was developed with an iterative participatory approach with its first version available within 6 months and with high acceptance—95% of Basic Emergency Care Course participants felt that it was useful. Our solution had minimal direct costs and resulted in a robust infrastructure for subsequent assessment and maintenance and allows for efficient feedback and expansion.

**Conclusions:**

We believe that utilizing Lean software development strategies may help global health advocates and researchers build eHealth solutions with a process that is familiar and with buy-in across stakeholders that is responsive, rapid to deploy, and sustainable.

## Introduction

### eHealth in Low- and Middle-Income Countries

Health systems in low- and middle-income countries continue to face considerable challenges in providing high-quality affordable accessible care [1]. Simultaneously, they have experienced an unprecedented increase in the number of users of mobile phone and internet technologies, as well as a decline in the price of devices and services [2]. Furthermore, most medical professionals report that smartphone and mobile technologies are useful for training and education [3]. As a result, many health program implementers and policy makers are exploring the extent to which eHealth, defined as “the use of information and communication technologies for health” [4], can help address the challenges faced by resource-constrained health markets in terms of the availability, quality, and financing of health care [5-10].

However, few of these initiatives succeed as hoped or progress past the pilot phase. Although eHealth has the potential to strengthen health systems worldwide, consistent use is extremely rare and the evidence base is immature [11]. Consequently, the opportunities to advance knowledge remain limited in scope [12]. Limitations in adoption are likely multifactorial, but they often fail to address the needs of diverse stakeholders [13]. eHealth apps often have unique end-users, and health care scientists and researchers are not often well-versed in how software engineers operate as traditional scientific measures may be too rigid to identify the nuanced differences between user subgroups [14]. Lastly, software and apps are expensive to develop, averaging between a cost of US $23,000-28,000 (with minimal features) to $270,000 (for robust apps)—a cost many community-based projects simply cannot afford [15-17].

These limitations in software development are not new, however. Early in the history of software product development and the expansion of the internet, there was concern over excessive amounts of money being spent to develop technologies that did not meet user needs. Years were sometimes spent building apps which would later be found to have been based on false assumptions, were uselessly developed for an outdated technology, or the demand for a solution disappeared by the end of the software production cycle. Development cycles were too long and rarely had early stakeholder buy-in or feedback. In response, developers looked for strategies to remain responsive to an ever evolving market while building solutions as quickly and efficiently as possible.

One of the most commonly employed methods evolved from the Toyota Production System, later coined as Lean, a systematic method for waste reduction [18]. The aim of employing Lean in software development was to use this approach to build solutions that created value for customers, eliminated waste, empowered the developers, and allowed for continuous improvement. Not only has Lean methodology become commonplace in software development, its use has been growing in global health care and as a validated method to employ evidence-based practices and continuous quality improvement in diverse of practical settings [18-20].

### Basic Emergency Care

The 2015 Global Burden of Disease Study [21] found that emergency medical diseases contributed to more than half of all years of life lost globally. While this continues to be a global burden, it is most acutely felt by low- and middle-income countries, which have 4.4 times the disease burden of high-income countries [21]. Moreover, it is estimated that over half of all deaths in these countries are potentially addressable by emergency care [22]. Well-organized emergency care systems play an important role in the delivery of emergency services to and the health outcomes of patients in low-resource settings [23]; however, frontline providers in low- and middle-income countries often lack the basic training to recognize and treat life-threatening conditions [24], and the accessibility of adequate emergency care in low-resource settings is limited [25]. The burden of acute diseases, coupled with the lack of emergency care training in low- and middle-income countries, begets unnecessary mortality.

Prioritizing an integrated approach to early recognition and resuscitation substantially reduces morbidity and mortality associated with emergent medical conditions [26,27]. As such, the World Health Organization developed an open-access Basic Emergency Care course to provide standardized training in basic assessment and life-saving techniques using a traditional lecture format. The course covers the Airway, Breathing, Circulation, Disability, and Exposure approach to pediatric and adult emergencies, trauma, respiratory distress, shock, pregnancy, and altered mental status. The Basic Emergency Care course is not, in itself, an eHealth intervention. It is traditional didactic training, consisting of a 5-day series of classes, aimed at teaching participants how to recognize and intervene in a number of emergent conditions (the most significant causes of morbidity and mortality

Participants are trained to conduct a primary survey of emergency situations they may encounter situation, using the mnemonic *Airway, Breathing, Circulation, Disability, and Exposure*, which is, in itself, a concise and standardized algorithm; however, many branch points and deviations as well as condition-specific interventions are possible. But while there are a large number of emergent medical conditions annually, each practitioner may only encounter a few of any particular type in a given year. Diagrammatic representations of the possible emergency situations that one might encounter contained multiple decision points and branches; thus, their complexity made them too unwieldy to be useful. Furthermore, not all emergent conditions take place in a hospital, with many occurring in the field and being managed by emergency medical technicians. Thus, a mobile point-of-care reference was suggested as a means to help individuals recall Basic Emergency Care course training.

**Figure 1:**
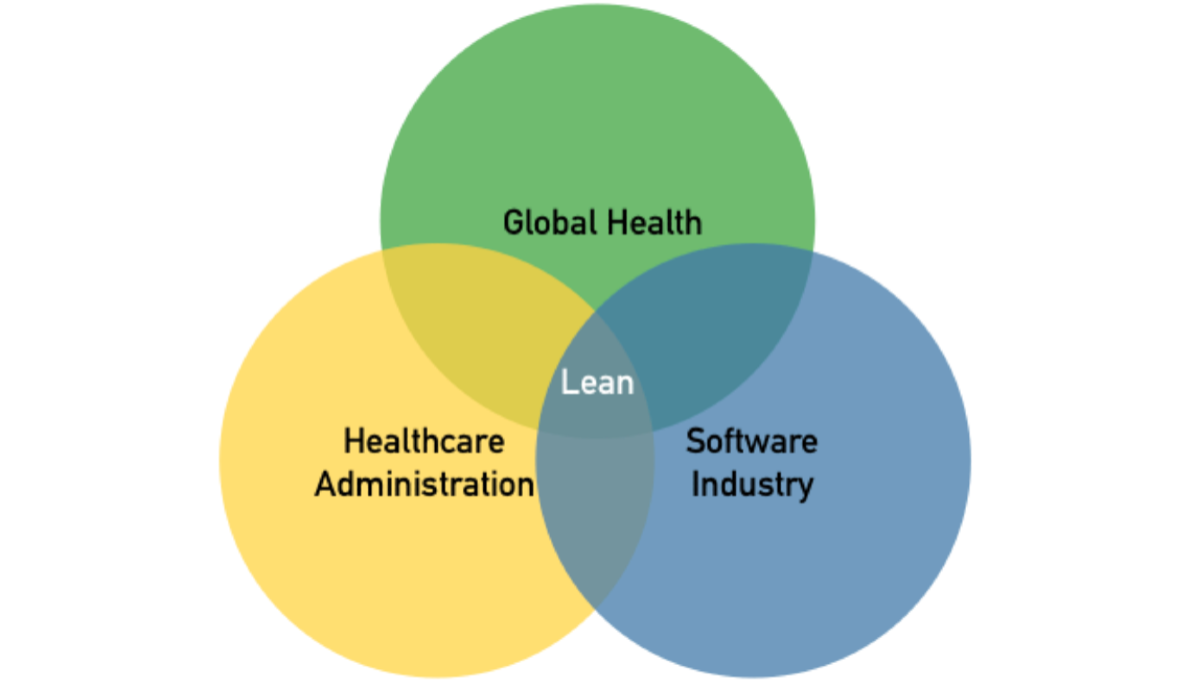
Stakeholder overlap with Lean methodologies.

While there are many software development models to choose from, such as Waterfall or Scrum, we believe that the breadth of use and familiarity of Lean across health care administration, the software industry, and global health make it the ideal model to be used to address some of the inherent difficulties of global health software development and implementation (Figure 1) [28,29]—each is necessary for developing eHealth interventions and each has robust familiarity with this methodology [19,30,31]. Global health experts and their stakeholders are necessary to understand the landscape of issues needing to be addressed within a community. Software developers need to be able to communicate with global health experts to understand those needs and the scope of technological solutions. Finally, health care administrators must be able to respond to their community and clinician needs as well as implement the developed solutions to work within their institution.

Given that the Lean process makes use of limited resources, empowers local stakeholders, and facilitates remote development, it may prove to be a successful model for wide-scale implementations [30]. We aimed at employing a standardized Lean software development strategy to quickly develop and iterate a community-based global eHealth initiative for diverse stakeholders. 

## Methods

### Lean Strategy

**Figure 2:**
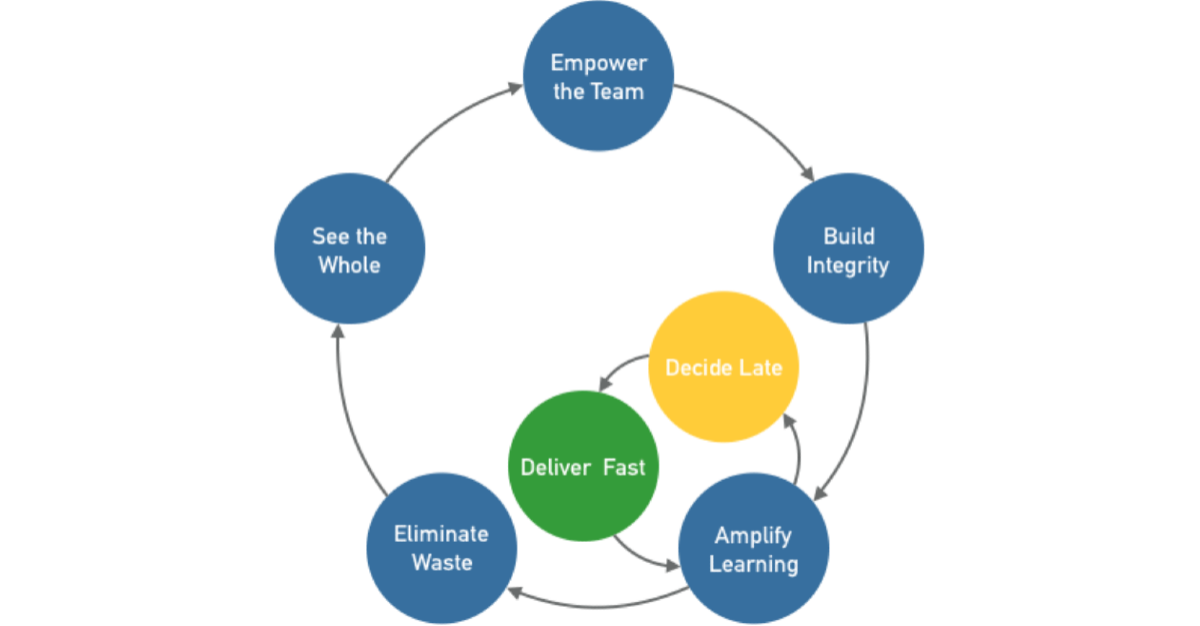
Lean workflow in global eHealth software development.

Lean software development has 7 core elements [32]: seeing the whole, empowering the team, building integrity in, amplifying learning, deciding as late as possible, delivering as fast as possible, and eliminating waste (Figure 2).

#### Seeing the Whole

**Figure 3:**
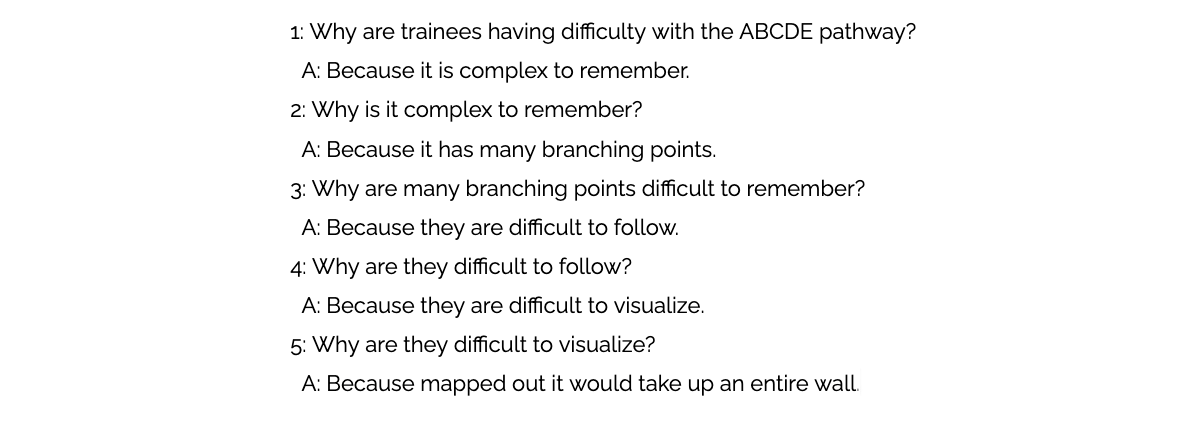
The 5 Whys for the Basic Emergency Care app.

Complex systems are composed of networks of interconnected components that influence each other, often in a nonlinear fashion [33]. Health care, specifically global health care, is a complex system. Problems are rarely simple and rarely have clear boundaries [34,35]. In order to develop broad and novel solutions, teams must be able to see the whole and avoid a hammer-looking-for-nails approach. In global health care, this is often exacerbated by a tendency to focus on squeaky wheels [36] that distract from the true underlying issue—which may be more difficult to address. To address this problem, Lean systems use root-cause analyses. While there are many different types of analyses, one simple approach is called *The 5 Whys* (Figure 3) [37,38]. This is an iterative interrogation technique used to explore the cause-and-effect relationships underlying a particular problem with the goal of determining the root cause by repeating the question “Why?”

Initial project planning using this approach began in early 2016 with input from health care providers and software developers. Because groups tend to overestimate the importance of their particular skill set, solutions to a root-cause analysis must be derived from stakeholders without a priori assumptions. For the Basic Emergency Care app, it was participants who referenced their access and regular use of mobile phones for medical purposes which guided the solution.

#### Empowering the Team

Team empowerment strategies have been shown to improve many elements of health care, from individual management of disease, public health strategies, or improving partnership and sustainability in global health [39,40]. Team empowerment requires: (1) enhancing meaningfulness of work, (2) fostering participation in decision making, (3) facilitating goal accomplishment, and (4) providing autonomy and freedom from bureaucratic restraints that can cause unnecessary delays and bottlenecks [41].

For the Basic Emergency Care app, team empowerment began in mid-2016 with a shared vision of both the problem and solution. Developers, who lacked the experience of emergency department care or global health initiatives, spent time with those who had, in the form of structured meetings and ongoing email communication, throughout the app development and testing process. Efforts were made to make videoconferencing available to developers whenever possible during testing so that helpful features and hurdles to use could be identified and addressed in real time. Participation across the development team was fostered through a variety of free and secure resources, such as Slack (a collaboration hub for projects which allows document sharing and conferencing) and GitHub (a version control system widely used in software engineering which allows for shared to-do lists and assignment of tasks). By fostering shared decision making through open communication and regular feedback, team members were able to develop a plan for addressing their tasks, which was then shared with the team, and suggest changes as necessary based on their role in the team. This allowed for participation in decision making and autonomy in areas of subject-matter expertise.

#### Building Integrity In

**Figure 4:**
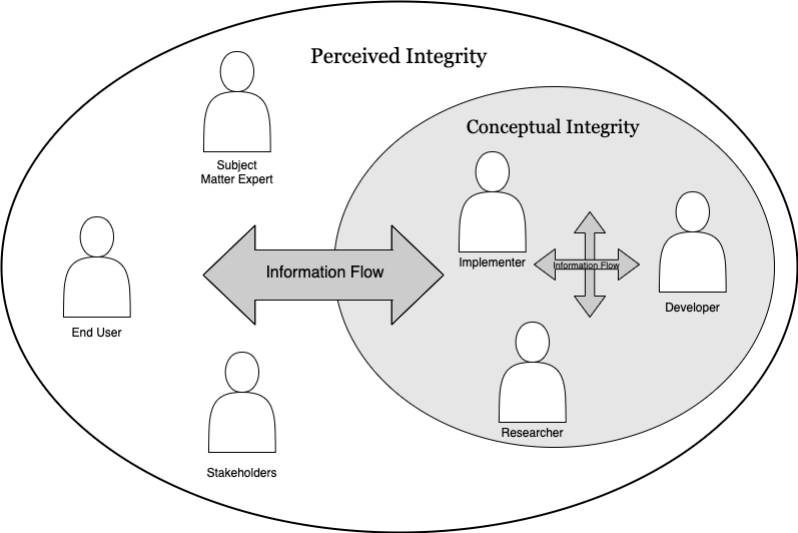
Integrity through information flow [30].

In the software engineering, *integrity* is described as a freedom from flaw, defect, or decay [42]; a solution should have internal and external consistency. That is, it should make intuitive sense to the end user how to interact with the solution (ie, perceived integrity), and the internal functioning of the solution should be the appropriate use of engineering and organization resources (ie, conceptual integrity). Software is usually expected to evolve gracefully as it adapts to the future. Solutions with integrity have a coherent architecture; score highly on usability and fitness for their purpose; and are maintainable, adaptable, and extensible. Research has shown that integrity comes from wise leadership, relevant expertise, effective communication, and healthy discipline [32]. Thus, integrity is achieved through excellent detailed information flow—from users to the development team and between the development team members (Figure 4) [43].

Achieving integrity for the Basic Emergency Care app first required an empowered team capable of communicating and receiving feedback as described above. During app development from September 2016 through January 2017, all team conversations and amendments to software were recorded using Slack and GitHub, allowing for easy sharing with new members as they joined the team smoothing onboarding and preventing delays when team members transitioned. Furthermore, feedback channels for remote team members and site leads or prototype testers were maintained to easily provide feedback and suggestions to optimize the solution’s fit.

#### Amplifying Learning

Gathering information is an important step in solving new problems. For complex problems, the preferred approach to a solution is to use the scientific method: observe, create a hypothesis, devise an experiment to test the hypothesis, run the experiment, and see if the results are consistent with the hypothesis [32,44]. Today it is widely accepted that software design is a problem-solving process that involves discovering solutions through short repeated cycles of investigation, experimentation, and results validation. In Lean development, this is considered the sum of 2 other steps—waiting to as long as possible to finalize a decision or acting quickly and soliciting feedback.

Learning from past interventions has been difficult in prior eHealth initiatives. This is sometimes due to rigid statistical measures that attempt to measure the effect of the software itself rather than how it corresponds to the principles on which it is based [45]. Furthermore, many initiatives do not even make it past the pilot stages, thus limiting the broad awareness of their successes and failures due to publication bias [7,45]. Implementation science measures such as reach, effectiveness, or acceptance are rarely reported, making it difficult to learn from prior endeavors or predict successful strategies. Incorporation of theory-informed frameworks for understanding factors associated with adoption and nonadoption are essential for evaluating eHealth software and generating knowledge that can be applied to other settings [46].

Given that we expected he Basic Emergency Care app to be used in many different settings, we aimed to develop the app with avenues of concomitant feedback that would allow for product improvements and implementation improvements. This included readily available and standardized survey methods and a grounded theory approach to leading focus groups. We conducted these sessions, in Tanzania and South Africa from January 11, 2017 through February 5, 2017. Feedback from each session was used to modify the app; iterative changes to the app were tested in subsequent groups.

#### Deciding as Late as Possible

Development practices that provide for late decision making are effective in domains that involve uncertainty because they provide an option-based approach. A key strategy for delaying commitments when developing a complex solution is to build a capacity for change into the system. Delayed decisions are valuable because they can be made based on facts rather than speculation. This is also sometimes referred to as concurrent development or just-in-time production [47,48] Concurrent development allows for a breadth-first approach, to discover big costly problems early on in the development process (before it is too late). Concurrent development, as opposed to sequential development, means a team starts programming the highest value features as soon as high-level conceptual designs have been determined; detailed requirements may still be undergoing investigation. In addition to ensuring against costly mistakes, concurrent development is considered to be the best method for dealing with changing requirements [32]. This is because not only are the big decisions deferred while considering all options, but the little decisions are deferred as well. When change is expected, concurrent development reduces delivery time and overall cost, while improving the performance of the final product. But concurrent software development also has costs. It requires having developers with enough expertise in a particular domain (ie, mobile, machine learning, etc) to anticipate where the emerging design is likely to lead and having collaboration between customers and analysts [32].

During initial user feedback sessions for the Basic Emergency Care app, some participants suggested only allowing the user to progress linearly through the entire pathway thus forcing users to complete the entirety of the primary survey every time they use the app, but a particular subgroup requested the ability to simply search course material as soon as they identified a problem. Given that whether users would be allowed to jump through the app or progress linearly fashion would require significantly different interfaces and functionality (ie, a search bar and the ability to bypass certain checkpoints), it was necessary to test both options and delay decision, until the options had been vetted by domain experts or one had to be deployed. During the first iteration of this process in February 2017, engineers examined the product roadmap to determine whether this feature would even be possible, while end users provided feedback about feature necessity and value.

#### Delivering as Fast as Possible

While seemingly contradictory, *delivering as fast as possible* complements *deciding as late as possible*. The faster a change or feature can be delivered, the longer decisions can be delayed before testing. Furthermore, without speed, it is not possible to have reliable feedback. In software development, the discovery cycle—design, implement, feedback, improve—is critical for learning. The shorter these cycles, the more can be learned.

For the Basic Emergency Care app navigation, a software solution that made both options possible, was chosen early during app development (in late 2016). Team members tested this solution for bugs. Both options were later deployed to determine the impact of each option on user satisfaction, app use, or recall of the course material.

#### Eliminating Waste

As the name implies, eliminating waste is the core concept of the Lean development strategy. Waste can come in many forms; from monetary to time. Anything that does not directly add value to the end-user is a waste. There are some particular methods that have been described to see and eliminate waste in software development, called the 7 wastes: partially done work, extra processes, extra features, task switching, waiting, motion, and defects [49]. Eliminating waste is a central tenant of the Lean development strategy. Most authors suggest starting with an approach to eliminate waste. However, in global health, eliminating waste requires a holistic view of the problem, an understanding of what is possible, and stakeholder involvement as well as feedback before assumptions are made. Thus, we have this pivotal step at the end as a reminder to assess the prior work in context. Many of the strategies to eliminate waste are addressed by the other 6 core elements of Lean. Solutions with integrity limit motion and task-switching by allowing for bidirectional feedback from users to the engineering team so that workflows may be taken into account in the solution. Partially done work is limited when decisions are made only when absolutely necessary. Waiting is minimized by delivering as fast as possible. Defects are identified by amplifying learning. Finally, extra features and processes are only identified by seeing the whole and understanding the purpose or utility of each within the greater context of addressing the problem at hand.

**Figure 5:**
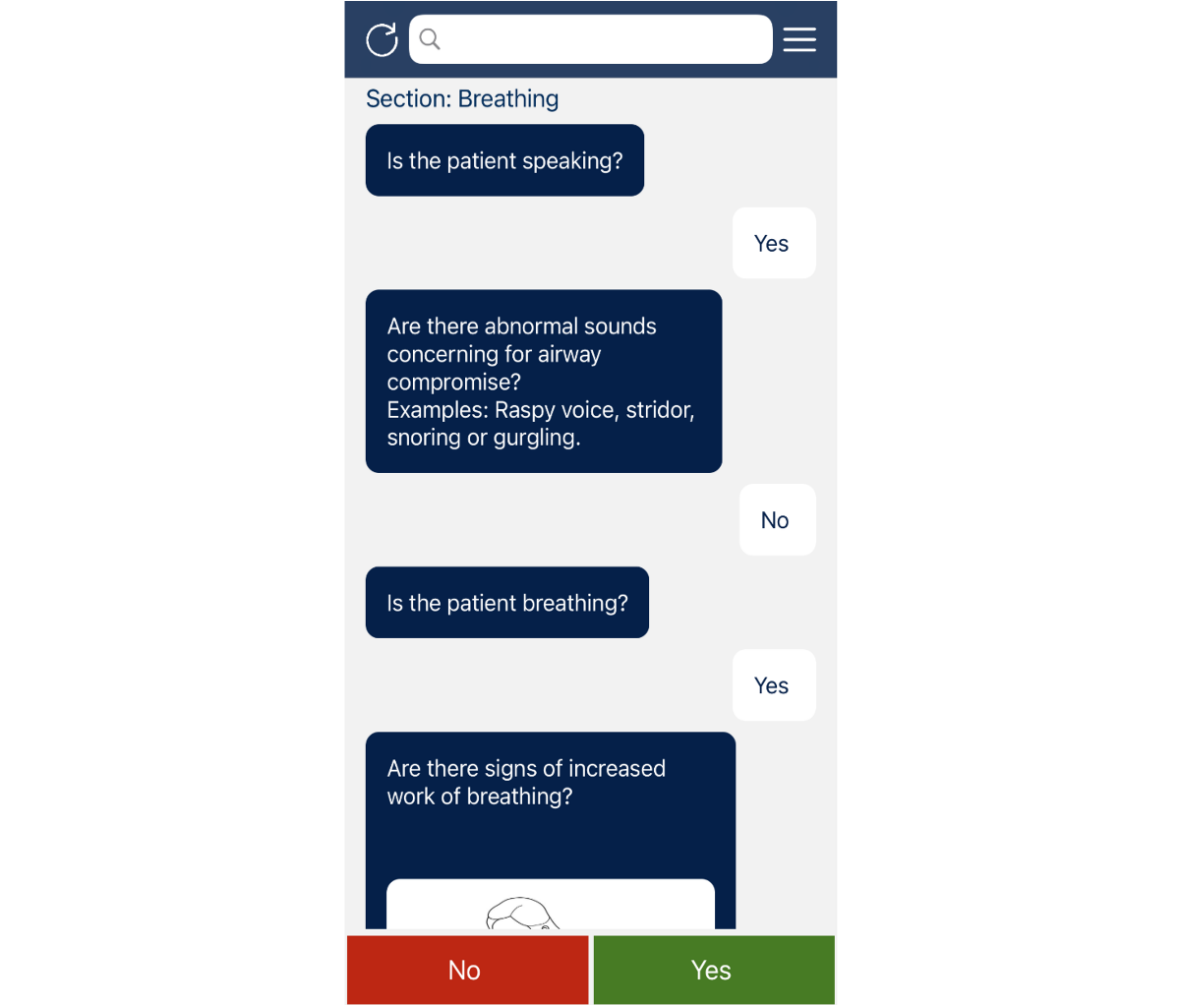
Screenshot of the Basic Emergency Care app.

For the Basic Emergency Care app, waste could be considered in 2 dimensions: cost and time. Waste in both of these dimensions could result from unnecessary features or delays in feedback or delivery of requested features. Thus, we utilized continuous feedback from stakeholders and iterative testing to ensure that we were not expanding the scope or necessary elements of our solution. This helped us to limit engineering demands and to give directed instructions to developers, which helped to limit costs and time. We were able to create our pilot app (Figure 5) for the cost of a single Android and iOS app developer (who was hired for a per-project fee of $3000) and web-hosting ($96 annually). Development took 6 months (from initial scope description until pilot completion).

## Results

In a convenience sample of participants from across all levels of training, the majority (56/59, 95%) believed that the Basic Emergency Care app was “useful for their practice.” Responses included that it was an effective means to help “remind (me) to complete the primary survey on all patients” and prevented “missing steps.” A subgroup of physicians trained in emergency medicine (9/30, 30%) reported that they would prefer if the tool allowed “searching conditions” or references for “specific interventions” rather than a rigid algorithm as “I already know what the problem is, but now I want to know how to address it.” This feedback was used to design the subsequent versions of the app to improve acceptance and usability for all target providers.

## Discussion

### General

Our goal was to develop a mobile app through the use of a model that was well-known to each participant stakeholder in a global health initiative: global health, health care administration, and software engineering. We found that in this setting, the Lean software development model was effective for limiting costs, addressing stakeholder needs and local practices, and bringing solutions to scale quickly.

Online calculators estimated a cost between $11,700 to $71,000 to develop an app such as the Basic Emergency Care app with similar data and infrastructure needs [50,51]. However, we were able to build our pilot app for a fraction of that cost at total monetary cost of $3096 for software development and annual web-hosting. Of course, we recognize that there are other costs associated with development that must be taken into account. While the monetary cost of our app was low, it took much longer to develop than the estimates given for professional app development. But we believe that this speed was likely balanced by the benefit of continuous feedback in a participatory model which helped to empower the team and build integrity [52-55]. The participatory model emphasized by the Lean software development model is particularly helpful in global health app development where needs span geographic, linguistic, and technological barriers.

There is a poignant need for mobile solutions in low- and middle-income countries. Many health care providers there have mobile devices, and there has been an increase in the development of apps to support care in these locations [2]. However, global health systems are complex and have different practices, system needs, and user demographics [13]. Thus, meaningful resources should not be built entirely external to the primary stakeholders, as many have been to date. However, access to software engineers can be limited for global health interventions that do not often have long-term scalability, need for full-time engineering services, or typical funding. When professional engineering services are employed, time with the engineers is often limited, due to cost or access, and familiarizing them with local needs can take time, limiting their contributions.

However, we do not believe this must necessarily be the case. Given that the Lean model allows for robust stakeholder involvement, rapid iteration, and a holistic approach to problems while sharing a common language for global health, software development, and health care administration, we believe that it should be the model of choice for software development in low- and middle-income countries.

### Limitations

This study had several limitations that need to be considered. First, the communities of Tanzania and South Africa may not be representative of other low- and middle-income countries outside of sub-Saharan Africa. The goal is for the Basic Emergency Care app to be available worldwide, though it is currently only available in English-speaking countries. However, part of utilizing a model such as Lean is to promote the practice and skills necessary to generalize solutions to any community. We believe that the process of *seeing the whole*, in conjunction with the other core elements, make this model particularly well-suited to communities that may be different from those in which we tested our model and software.

Additionally, while our primary university affiliation is in the heart of a major metropolitan software and technology development area (ie, Silicon Valley), other programs may not find it as easy to engage a rounded team of health professionals, engineers, and stakeholders locally in their community. The Lean iterative process requires the timely engagement of team members to effectively suggest, implement, and revise eHealth software. This can be especially challenging when team members operate in different time zones and with different cultural backgrounds. However, as training and education for computer engineers continues to increase, with accessibility in-person and online, there continues to be growth of local engineering capabilities across the globe. The model utilized above is meant to be agnostic to the problem being addressed and the particulars of coding languages.

Similarly, new software development strategies, such as Scrum, continue to gain popularity, and newer models evolve all the time. It’s possible that these strategies will prove to be even more efficient than Lean for producing and iterating software products. However, Lean has gained broad acceptability in a multitude of industries across borders and specialties since it was first introduced. This makes it particularly well-suited to maintaining the robust source of knowledge and integrity necessary for consistent global health interventions.

### Conclusion

Lean processes have become a standard in the software development industry, but the methodology also has a proven track and has gained broader acceptance in the health care administration and global health communities. Thus, the Lean methodology presents a particularly suited framework which is already available to most of the parties involved in developing global eHealth solutions. Furthermore, its ideals—such as empowering the team, eliminating waste, and having a big-picture view, are implicitly aligned with those of global health interventions. We believe Lean strategies applied to software development for global health initiatives, particularly for low- and middle-income countries, may address some of the concerns regarding the prior limitations of these interventions.
